# Nomogram Based on Log Odds of Positive Lymph Nodes Predicting Cancer-Specific Survival in Patients With T3 and T4 Gallbladder Cancer After Radical Resection

**DOI:** 10.3389/fsurg.2021.675661

**Published:** 2021-10-27

**Authors:** Chen Yuan, Qiaomeng Tao, Jian Wang, Kai Wang, Shubing Zou, Zhigang Hu

**Affiliations:** ^1^Department of Hepatobiliary Surgery, The Second Affiliated Hospital of Nanchang University, Nanchang, China; ^2^Department of General Surgery, The Second Affiliated Hospital of Nanchang University, Nanchang, China; ^3^Department of Oncology, The Second Affiliated Hospital of Nanchang University, Nanchang, China

**Keywords:** gallbladder cancer, nomogram, prognostic, cancer-specific survival, LODDS

## Abstract

**Background:** The aim of this study based on log odds of positive lymph nodes (LODDS) is to develop and validate an effective prognostic nomogram for patients with T3 and T4 gallbladder cancer (GBC) after resection.

**Patients and Methods:** A total of 728 T3 and T4 gallbladder cancer patients after resection from the Surveillance, Epidemiology, and End Results (SEER) database, randomly divided into training cohort and validation cohort according to 7:3. Another 128 patients from The Second Affiliated Hospital of Nanchang University for external validation. The nomograms were built by the Cox regression model and the Fine and Grey's model. Concordance index (C-index), calibration curve and the area under receiver operating characteristic (ROC) curve (AUC) were used to evaluate the nomogram and internal verification. The decision curve analysis (DCA) was used to measure clinical applicability.

**Result:** LODDS was independent prognostic predictor for overall survival (OS) and cancer-specific survival (CSS), and established the nomograms on this basis. The nomogram we have established has a good evaluation effect, with a C-index of 0.719 (95%CI, 0.707–0.731) for OS and 0.747 (95%CI, 0.733–0.760) for CSS. The calibration curves of OS and CSS both showed good calibration capability, and the AUC for predicting 1-, 2-, and 3-year 0.858, 0.848 were and 0.811 for OS, and 0.794, 0.793, and 0.750 for CSS. The DCA of nomograms both showed good clinical applicability.

**Conclusion:** The nomogram can provide effective OS and CSS prediction for patients with advanced gallbladder cancer after surgery.

## Introduction

Gallbladder cancer (GBC) is a malignant tumor formed from the mucosa of the gallbladder (1). GBC is relatively common in biliary malignancies, accounting for 80–95% of these patients (2). The prognosis of GBC is extremely poor, with an overall 5-year survival rate of <5% (3, 4), due to the lack of obvious typical clinical symptoms and early diagnostic strategies (5). Surgical resection is still the best treatment strategy for long-term survival, and systemic treatment is very important for advanced stage patients (6). Since GBC is prone to recurrent even after complete surgical resection (7, 8), radical resection supplemented with radiotherapy and chemotherapy is essential (9). Besides, lymph node metastasis is the most common way of metastasis in GBC, the lymph node stage and dissection serve as important roles affecting the prognosis of surgery (10, 11). Therefore, an accurate GBC prognostic model is very important for clinicians, which can provide individualized treatment strategies for high-risk patients.

The AJCC 8th TNM staging system is the most commonly used staging system for GBC, an N staging divides N1 and N2 by the number of metastatic lymph nodes. Recent study showed that positive lymph node ratio (pLNR) is more accurate to predict the prognosis of GBC (12). Besides, some other studies have proved that log odds of positive lymph nodes (LODDS), which was defined as the ratio of the number of positive lymph nodes to the number of negative lymph nodes, may have a better predictive effect (13). Many studies have shown that LODDS has a good predictive effect on cervical cancer (14), ovarian epithelial cancer (15), and gastric cancer (16). With age increased, most patients face comorbidities, and many patients die from non-tumor causes, which also causes competitive mortality (17). Therefore, we need to take these factors into consideration in order to avoid errors (18). On this basis, we developed new predictive nomograms to predict the overall survival (OS) and cancer-specific survival (CSS) of patients. Surveillance, Epidemiology, and End Results (SEER) database is an open database from which we collected data for research.

## Patients and Methods

### Study Design

We retrospectively collected T3 and T4 gallbladder cancer patients after resection from the Surveillance, Epidemiology, and End Results (SEER) database, randomly divided into training cohort and validation cohort according to 7:3. Another patients from The Second Affiliated Hospital of Nanchang University for external validation. Through analysis, the independent risk factors that affect the patient's OS and CSS were obtained, and nomograms were constructed based on these. The aim of this study based on log odds of positive lymph nodes (LODDS) is to develop and validate an effective prognostic nomogram for patients with T3 and T4 gallbladder cancer (GBC) after resection.

### Patient Enrollment

Patients pathologically diagnosed with advanced gallbladder cancer and underwent radical surgery were retrospectively collected. The training and internal verification cohorts came from the Surveillance, Epidemiology, and End Results (SEER) database for the period of 2010–2015 according to ICD-O-3/WHO 2008 classifications. The external verification cohort came from The Second Affiliated Hospital of Nanchang University for the period of 2010–2018. Radical surgery was defined as follows: (1) Hepatectomy: Gallbladder bed resection (>3 cm) or S4b+5 resection were routinely applied. Specially, hemihepatectomy plus pancreaticoduodenectomy or vessel reconstruction was considered if ligamentum hepatoduodenale was invaded; (2) Lymphadenectomy: N2 resection (No. 12/8/9/13); (3) Bile duct resection: Extrahepatic bile duct resection was considered if cystic duct was invaded. The exclusion criteria were as follows: (1) multiple primary tumors; (2) age <18 years; (3) incomplete follow-up data; (4) post-operative pathology suggested non-gallbladder adenocarcinoma; (5) survival time <1 month after surgery. Patients from SEER database were randomly divided into the training cohort and the internal verification cohort at a ratio of 7:3. This study was reviewed and approved by the ethics committee at our Institute and complied with the standards of the Helsinki Declaration and current ethical guidelines. Informed consent was exempt because it was accomplished retrospectively and anonymously. The flow chart is as follows:

### Statistical Analysis

In our study, cancer-specific death, and non-cancer-specific death were two competing events. The comprehensive impact of variables on total mortality and cancer-specific mortality is assessed by proportional hazard analysis with Fine and Grey's model (19). A nomogram was established based on independent risk factors extracted by multivariate analysis. The Kaplan-Meier method was used to analyze OS and CSS, and a long-rank test was used to compare the survival difference. Cox multivariate analysis was used to determine independent risk factors. The C-index and the calibration curve were used to evaluate the accuracy of the nomogram. In addition, we had also drawn the receiver operating characteristic (ROC) curve and the area under the curve (AUC) to evaluate the accuracy of the nomogram. By calculating the net benefit at each risk threshold probability, a decision curve analysis (DCA) is used to identify and compare the clinical value between the nomogram model and other clinical features (20–22). R Studio 1.3.1,056 was used to perform all the statistical analyses. *P* < 0.05 was considered statistically significant, and all tests were two-sided.

### The LODDS System

The logit of the positive lymph nodes, i.e., the LODDS, was defined as the log of the ratio between the probability of being positive lymph nodes and the probability of being negative lymph nodes when one lymph node is harvested. The definition of LODDS is log [(the number of positive lymph nodes +0.05)/(the number of negative lymph nodes +0.05)], where the pond is the number of positive lymph nodes and tnod is the total number of lymph nodes harvested, and 0.05 is added to both numerator and denomination to avoid the singularity. In our study, the LODDS ranged from −2.382 to 2.417. Therefore, we artificially divided the value of LODDS into four levels: LODDS1 (LODDS < -1.3), LODDS2 (−1.3≤ LODDS<0), LODDS3 (0≤ LODDS<1.3), and LODDS4 (LODDS≥1.3).


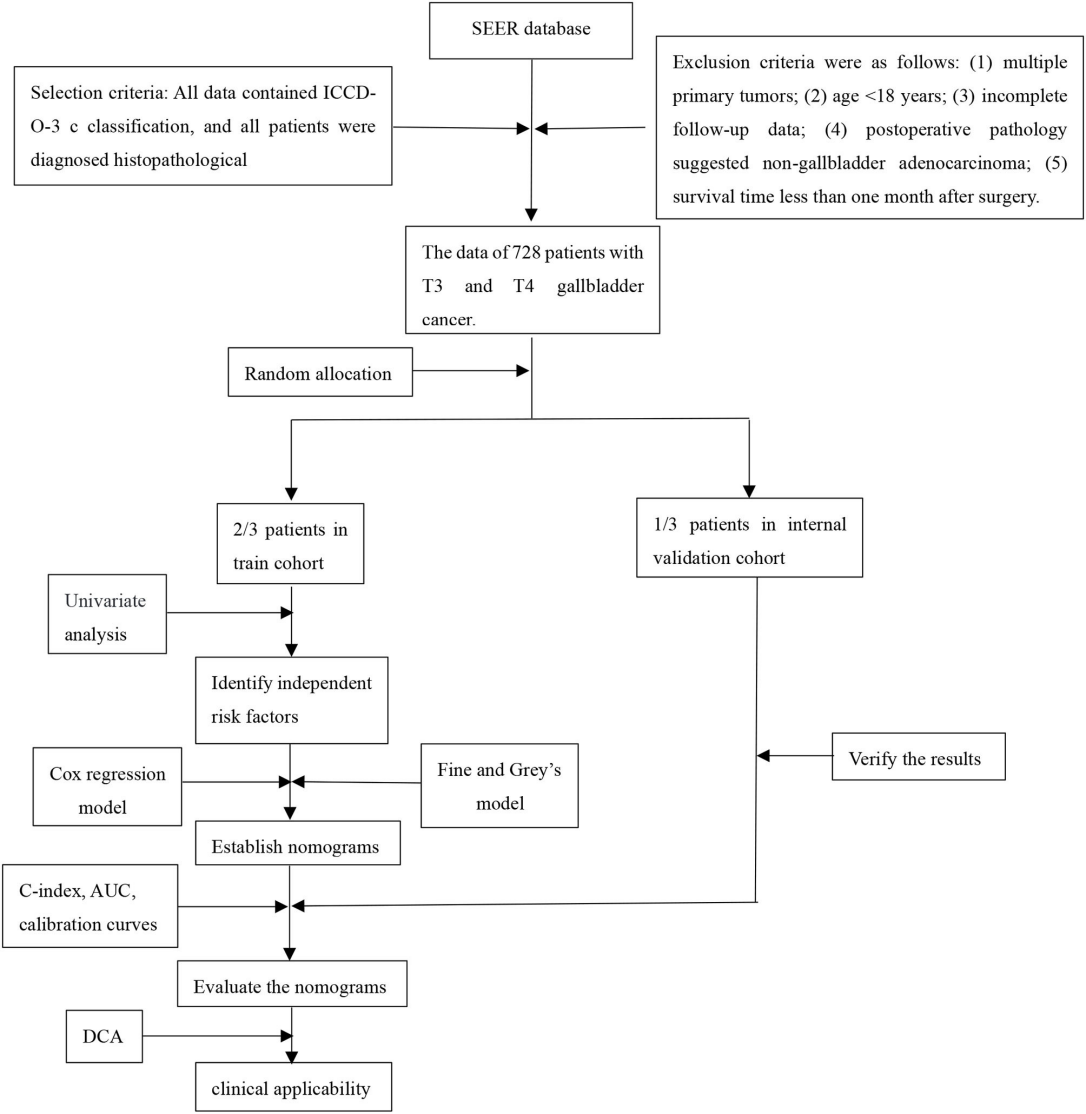


## Results

### Patient Characteristics

In this study, we collected a total of 684 patients diagnosed with advanced gallbladder cancer who underwent radical surgery from 2010 to 2015 from the SEER database and another 128 patients with advanced GBC after surgery from The Second Affiliated Hospital of Nanchang University for the period of 2010–2018. All patients were considered to be diagnosed as gallbladder cancer before surgery, followed by radical resection, and pathologically diagnosed as gallbladder cancer after surgery. Overall, 72.4% (495/684) of the patients were female, and 59.9% (410/684) of patients were older than 65 years. The grade of a little more than half patients (50.9%) was poor. A large scale of patients (76.3%) had not received radiotherapy, while a considerable number of patients (52.2%) received chemotherapy. A total of 43.7% (299/684) patients had lymph node metastasis, and the number of patients in LODDS level 2 was the largest.

**Figure 1:**
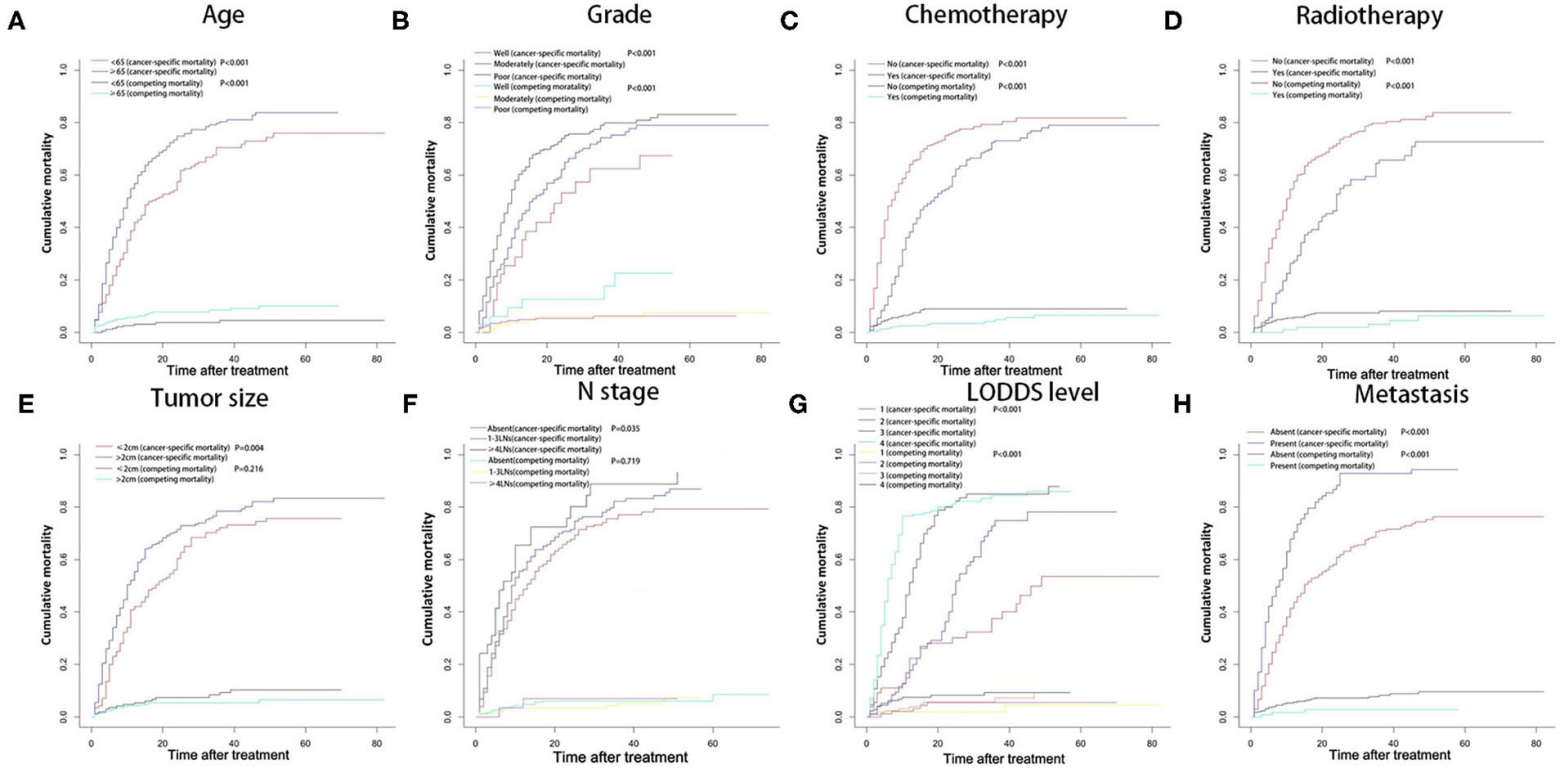
Cumulative cancer-specific and competing mortality according to patient characteristics: **(A)** age; **(B)** tumor grade; **(C)** chemotherapy; **(D)** radiotherapy; **(E)** tumor size; **(F)** N stage; **(G)** LODDS level; **(H)** metastasis. LODDS, log odds of positive lymph nodes.

A total of 540 patients died in our study, with the follow-up time ranged from 1 to 82 months, and the median time is 10.5 months. Five hundred one patients were cancer-specific death, and 39 died of other causes. With the increase of age, the cumulative death rate of GBC had gradually increased. The 1-, 2-, and 3-year overall survival rates, cancer-specific and non-cancer-specific survival were summarized in Table 1. The cumulative cancer-specific and competing mortality curves are showed in Figure 1. Older patients have significantly higher mortality rates than younger patients. Patients were more likely to die of GBC, but not other causes when the following characteristics were satisfied: older age, poorer grade of tumor differentiation, not performed radiotherapy or chemotherapy, distant metastasis and higher LODDS levels. As for the feature of tumor size, patients with larger tumors have a higher probability of dying from GBC, and there is no significant difference in competition risk.

**Table 1:** Overall survival rates and cumulative incidences of mortality among patients with gallbladder cancer.

**Characteristic**		**Patients**		**Overall survival rate (%)**			***P***	**Cancer-specific mortality (%)**			***P***	**Non-cancer-specific mortality (%)**			***P***
		**No**	**%**	**1-year**	**2-year**	**3-year**		**1-year**	**2-year**	**3-year**		**1-year**	**2-year**	**3-year**	
Total	684	100												
Age	<65	274	40.1	52.9	28.1	12.4	<0.001	40.1	55.5	65.3	<0.001	2.6	3.6	4.0	<0.001
	≥65	410	59.9	37.3	15.4	7.6		71.7	91.7	97.8		5.4	7.0	7.6	
Gender	Female	495	72.4	42.0	21.2	9.9	0.716	50.7	64.4	71.1	0.738	3.4	4.8	5.7	0.939
	Male	189	27.6	47.6	18.5	8.5		45.0	64.6	71.4		3.7	4.8	5.3	
Grade	Well	45	6.6	53.3	26.7	13.3	<0.001	33.3	53.3	62.2	0.001	6.7	8.9	8.9	0.029
	Moderately	291	42.5	50.9	25.8	11.3		41.2	57.4	66.3		2.7	4.5	4.5	
	Poor	348	50.9	36.2	15.2	7.5		57.8	71.8	76.4		3.7	4.0	4.3	
Tumor size	≤ 2 cm	264	38.6	48.5	25.4	9.8	0.010	44.7	59.1	69.3	0.004	3.0	4.5	4.9	0.216
	>2 cm	420	61.4	40.5	17.4	9.3		51.9	67.8	72.4		3.8	4.8	5.0	
Radiotherapy	No	522	76.3	34.7	14.0	5.9	<0.001	56.9	69.9	73.9	<0.001	4.4	5.7	5.9	<0.001
	Yes	162	23.7	72.2	41.4	21.0		24.1	46.9	62.3		1.2	1.8	3.0	
Chemotherapy	No	327	47.8	27.8	12.5	5.8	<0.001	62.4	72.8	74.9	<0.001	5.2	6.7	7.0	<0.001
	Yes	357	52.2	58.0	27.7	12.9		37.0	56.9	67.8		2.0	2.8	3.1	
LN metastasis	Absent	385	56.3	47.1	22.1	9.9	0.062	45.2	61.5	67.3	0.281	2.3	3.9	4.1	0.462
	Present	299	43.7	39.1	18.4	9.0		54.2	68.2	76.2		3.3	4.0	4.3	
N stage (8th)	Absent (M0)	385	56.3	47.1	22.1	9.9	0.019	45.2	61.5	67.3	0.048	2.3	3.9	4.1	0.752
	1–3 LNs (M1)	256	37.4	39.5	19.1	9.8		53.5	67.6	75.4		3.5	3.9	4.3	
	≥4 LNs (M2)	43	6.3	37.2	14.0	4.7		58.1	69.7	81.4		2.3	4.6	4.6	
Metastasis	Absent	509	74.4	49.3	25.0	11.8	<0.001	43.2	58.7	66.8	<0.001	41.	5.5	5.9	<0.001
	Present	175	25.6	26.9	7.4	2.9		66.3	81.1	84.0		1.7	2.3	2.9	
LODDS level	1	149	21.8	50.3	37.6	17.4	<0.001	39.3	60.7	64.6	<0.001	2.0	2.0	2.7	<0.001
	2	323	47.2	52.3	19.5	9.6		49.6	62.4	67.5		4.3	6.5	6.5	
	3	78	11.4	42.3	16.7	7.7		50.0	66.7	73.1		1.3	1.3	2.6	
	4	134	19.6	15.7	6.0	1.5		76.1	82.8	85.8		4.5	5.2	5.2	

*LN, lymph node; LODDS, log odds of positive lymph nodes, LODDS1 (LODDS < -1.3), LODDS2 (−1.3≤ LODDS <0), LODDS3 (0≤ LODDS <1.3), and LODDS4 (LODDS≥ 1.3)*.

**Figure 2:**
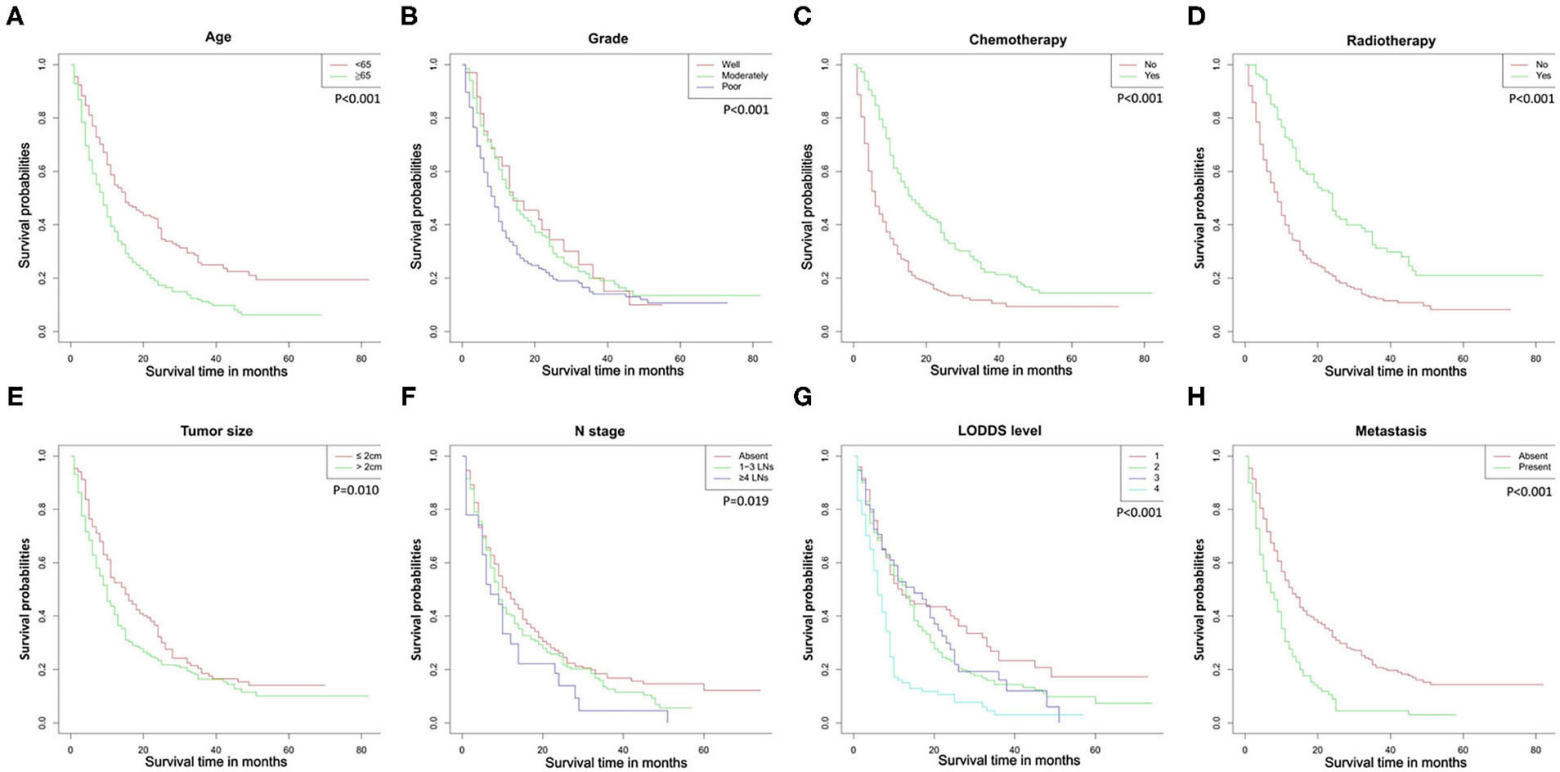
Overall survival rates according to patient characteristics: **(A)** age; **(B)** tumor grade; **(C)** chemotherapy; **(D)** radiotherapy; **(E)** tumor size; **(F)** N stage; **(G)** LODDS level; **(H)** metastasis. LODDS, log odds of positive lymph nodes.

The Kaplan-Meier curves used to compare OS were shown in Figure 2. Patients had significantly worse OS when the following characteristics were satisfied: older age, poorer grade of tumor differentiation, larger tumor size, not performed radiotherapy or chemotherapy, later N stage, distant metastasis, and higher LODDS levels.

### Univariate and Multivariate Analyses of Significant Prognostic Factors on OS and CSS

In our study, the median OS of patients was 10 months, and the 1-, 2-, and 3-year OS rates for all patients were 43.6, 20.5, and 9.5%, respectively. The univariate analyses of OS showed that age, LN (lymph node) metastasis, N stage, distant metastasis, tumor grade, tumor size, chemotherapy, and LODDS level had a significant impact on survival. In addition, the multivariate analyses of competing showed that age, LN metastasis, distant metastasis, tumor grade, tumor size, chemotherapy, and LODDS level also had a significant impact on cancer-specific survival. Gender showed no significant correlation with OS and CSS. All the results of univariate and multivariate analyses were shown in Table 2.

**Table 2:** Univariate and multivariate analyses of survival in patients with advanced gallbladder cancer.

**Characteristic**		**Overall survival**						**Cancer-specific survival**					
		**Univariate analysis**			**Multivariate analysis**			**Univariate analysis**			**Multivariate analysis**		
		**HR**	**95%CI**	***P***	**HR**	**95%CI**	***P***	**HR**	**95%CI**	***P***	**HR**	**95%CI**	***P***
Age (years)	<65/≥65	1.750	1.411–2.169	<0.001	1.416	1.138–1.761	0.002	1.496	1.203–1.859	<0.001	1.414	1.109–1.803	0.005
Gender	Female/Male	0.987	0.784–1.242	0.909			NI	1.032	0.816–1.307	0.789			NI
Tumor grade	Well/Moderately/Poor	1.497	1.371–1.628	<0.001	1.525	1.267–1.731	<0.001	1.548	1.197–1.849	<0.001	1.492	1.238–1.749	<0.001
Tumor size	≤ 2 cm/>2 cm	1.240	1.003–1.533	0.046	1.172	0.944–1.455	0.149	1.271	1.019–1.586	0.033	1.249	0.997–1.565	0.053
Radiotherapy	No/Yes	0.461	0.359–0.591	<0.001	0.665	0.498–0.891	0.005	0.493	0.383–0.636	<0.001	0.702	0.558–0.816	0.008
Chemotherapy	No/Yes	0.480	0.390–0.592	<0.001	0.542	0.425–0.693	<0.001	0.492	0.397–0.609	<0.001	0.691	0.533–0.895	0.005
LN metastasis	Absent/Present	1.214	0.990–1.490	0.049			NI	1.225	0.991–1.525	0.060			NI
N stage	Absent/1–3 LNs/≥4 LNs	1.552	1.030–2.336	0.035			NI	1.548	1.011–2.371	0.443			NI
Metastasis	Absent/Present	1.770	1.413–2.216	<0.001	1.774	1.402–2.244	<0.001	1.884	1.498–2.380	<0.001	1.612	1.216–2.136	<0.001
LODDS level	1/2/3/4	1.875	1.497–2.415	<0.001	1.740	1.047–2.891	<0.001	1.793	1.106–2.763	<0.001	1.748	1.247–2.594	<0.001

*LN, lymph node; LODDS, log odds of positive lymph nodes, LODDS1 (LODDS< -1.3), LODDS2 (−1.3≤ LODDS < 0), LODDS3 (0≤ LODDS< 1.3), and LODDS4 (LODDS ≥1.3). HR, hazard ratio; CI, confidence interval; NI, not included*.

The multivariate analysis of OS and CSS through the significant variables of univariate analysis showed in Table 2. Through multivariate analysis, the independent risk factors for OS were determined as age, distant metastasis, tumor grade, radiotherapy, chemotherapy, and LODDS level. In addition, the independent risk factors for CSS were determined as age, distant, tumor grade, chemotherapy, and LODDS level.

### Construction and Validation of Nomograms for OS and CSS

**Figure 3:**
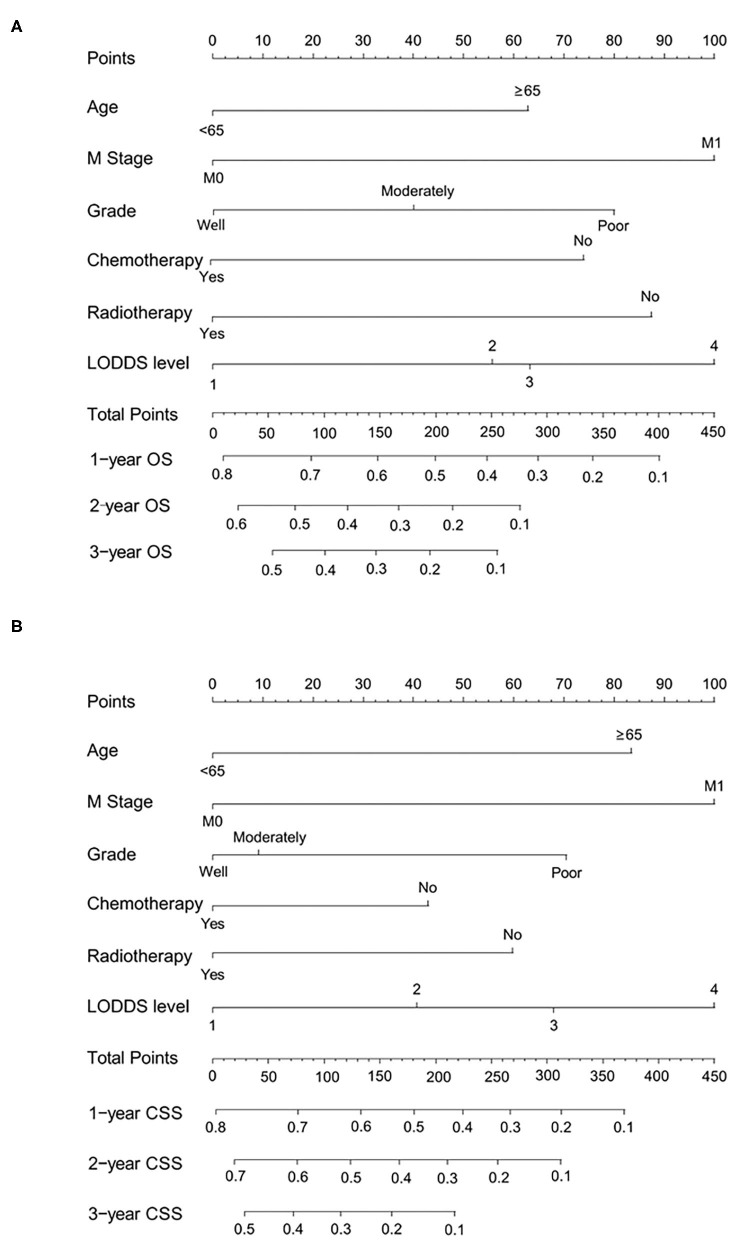
Nomograms predicting 1-, 2-, and 3-year OS **(A)** and CSS **(B)** of patients with advanced gallbladder cancer after resection. OS, overall survival; CSS, cancer-specific survival; LODDS, log odds of positive lymph nodes.

**Figure 4:**
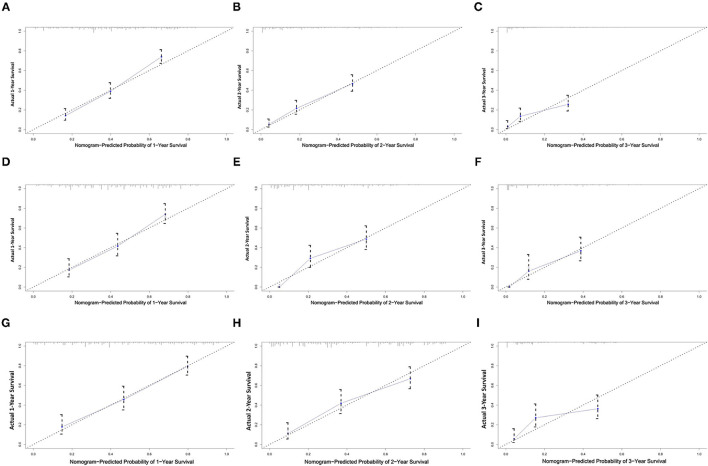
Calibration plots of the nomogram for 1-, 2-, and 3-year OS prediction of the training set **(A–C)**, internal validation set **(D–F)**, and external set **(G–I)**. X-axis represents the nomogram-predicted probability of survival; Y-axis represents the actual OS probability. A perfectly accurate nomogram prediction model would result in a plot that the observed and predicted probabilities for given groups fall along the line. Dots with bars represent nomogram-predicted probabilities along with 95% confidence interval.

**Figure 5:**
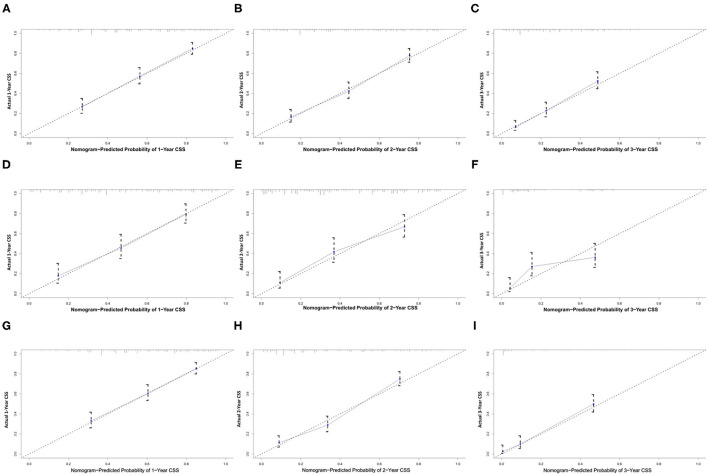
Calibration plots of the nomogram for 1-, 2-, and 3-year CSS prediction of the training set **(A–C)**, internal validation set **(D–F)**, and external set **(G–I)**. X-axis represents the nomogram-predicted probability of survival; Y-axis represents the actual CSS probability. CSS, cancer-specific survival.

All independent risk factors for OS and CSS were included in the nomogram. As shown in Figure 3, we used the training cohort to establish the nomograms predicting 1-, 2-, and 3-year OS and CSS rates. By adding up the scores of each selected variable, the probability of individual patient survival can be easily calculated. With a C-index of 0.719 (95%CI, 0.707–0.731), the nomogram showed good accuracy for OS prediction. The calibration curves of 1-, 2-, and 3-year OS probabilities showed that both in the training and validation cohorts, the survival rates predicted by the nomogram were consistent with the actual survival rates (Figure 4). In addition, the nomogram also showed good accuracy for CSS prediction, with a C-index of 0.743 (95%CI, 0.733–0.760). Calibration curves of CSS probabilities for 1-, 2-, and 3-year also showed the good agreement between nomogram predictions and actual observations in the training and validation cohorts (Figure 5).

**Figure 6:**
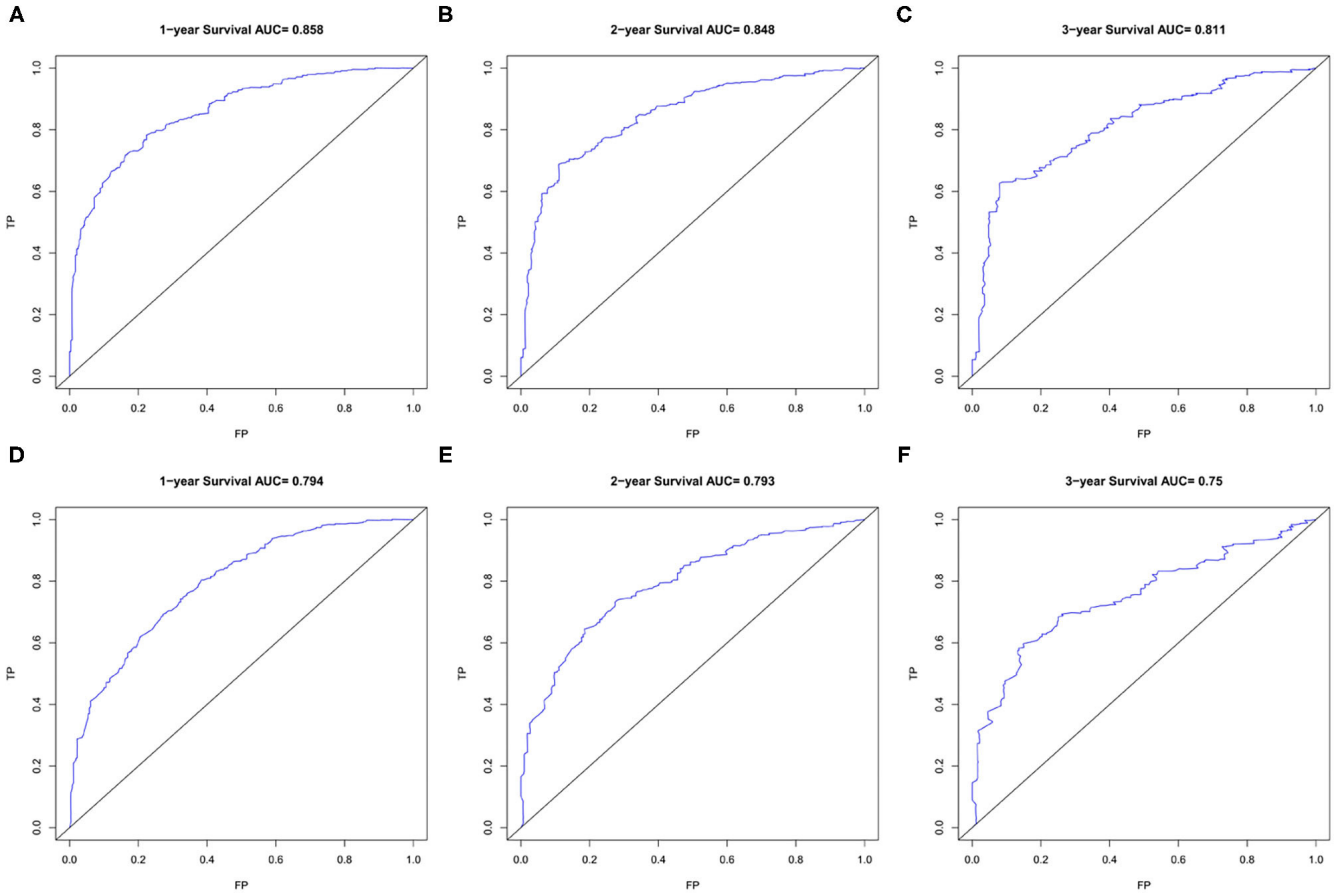
The ROC curves of the nomogram for 1-, 2-, and 3-year OS prediction **(A–C)** and CSS prediction **(D–F)**. OS, overall survival; CSS, cancer-specific survival.

**Figure 7:**
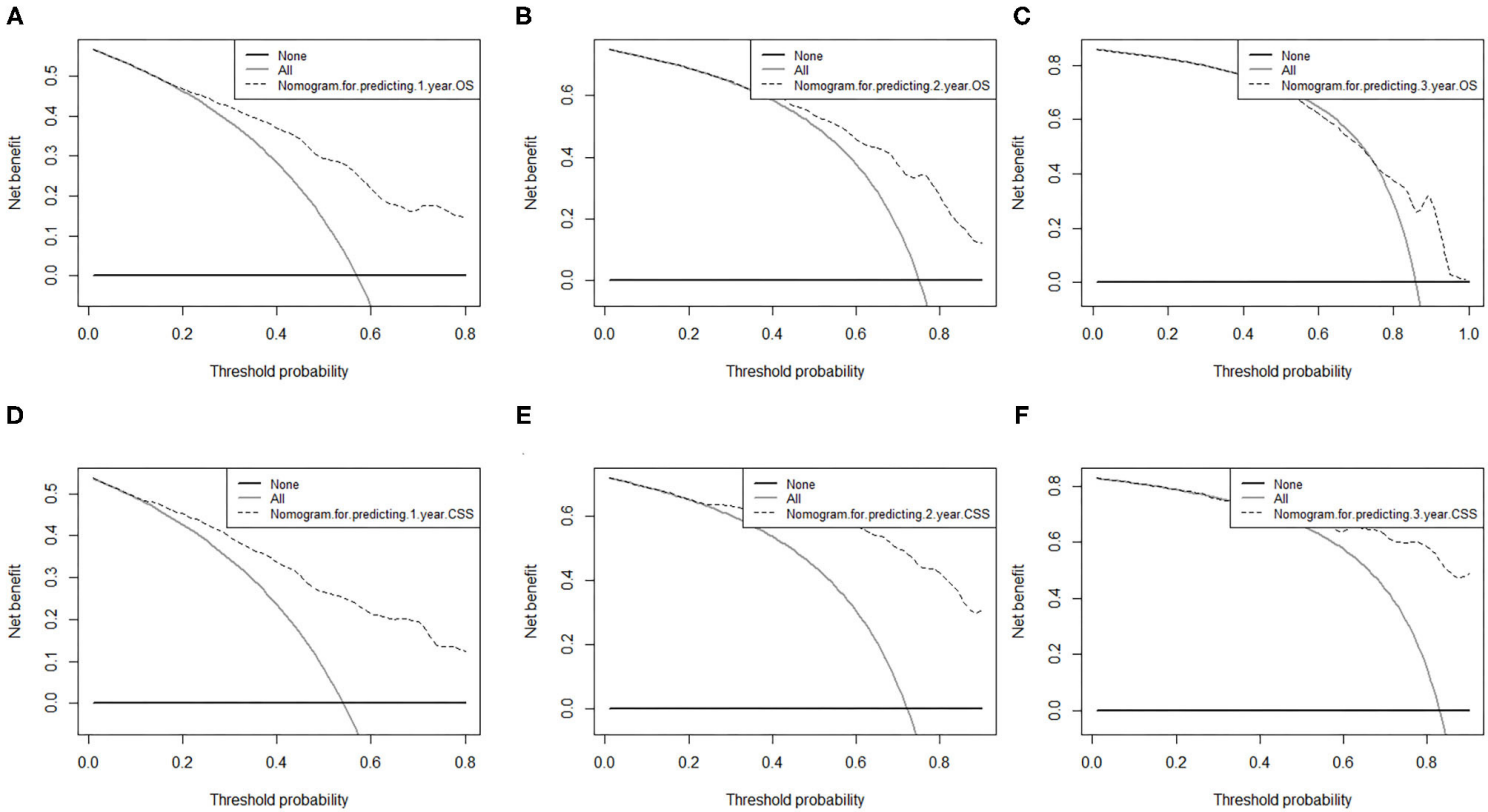
Decision curve analysis of nomograms for predicting **(A)** 1-year OS; **(B)** 2-year OS; **(C)** 3-year OS and **(D)** 1-year CSS; **(E)** 2-year CSS; **(F)** 3-year CSS. OS, overall survival; CSS, cancer-specific survival. The dashed lines indicate the net benefit of the models across a range of threshold probabilities. The horizontal solid black line represents the hypothesis that no patients experienced the endpoint, and the solid gray line represents the hypothesis that all patients met the endpoint. At different threshold probabilities within a given population, the number of high-risk patients and the number of high-risk patients with the outcome were plotted.

The area under ROC curve (AUC) values were used to predict the accuracy of nomogram. For the whole cohort, the AUC values of the nomogram for predicting the 1-, 2-, and 3-year OS rates were 0.858, 0.848, and 0.811, and in CSS were 0.794, 0.793, and 0.750 (Figure 6). AUC values were observed in the training cohort, internal validation cohort and external validation cohort were higher than 0.7 (Table 3). Finally, the decision curve analysis (DCA) was used to evaluate the clinical application value of the nomogram, which were evaluated by the threshold value of each DCA curves (Figure 7).

**Table 3:** The AUC value of nomogram for predicting 1-, 2-, and 3-year OS and CSS.

**Patients**	**Overall survival**			**Cancer-specific survival**		
	**1-year**	**2-year**	**3-year**	**1-year**	**2-year**	**3-year**
Training cohort	0.858	0.848	0.811	0.794	0.793	0.750
Internal validation cohort	0.841	0.839	0.818	0.801	0.784	0.739
External validation cohort	0.794	0.799	0.781	0.772	0.789	0.796

### Survival Analysis and Competing Risk Analysis Based on the Risk Stratification of the Nomogram

**Figure 8:**
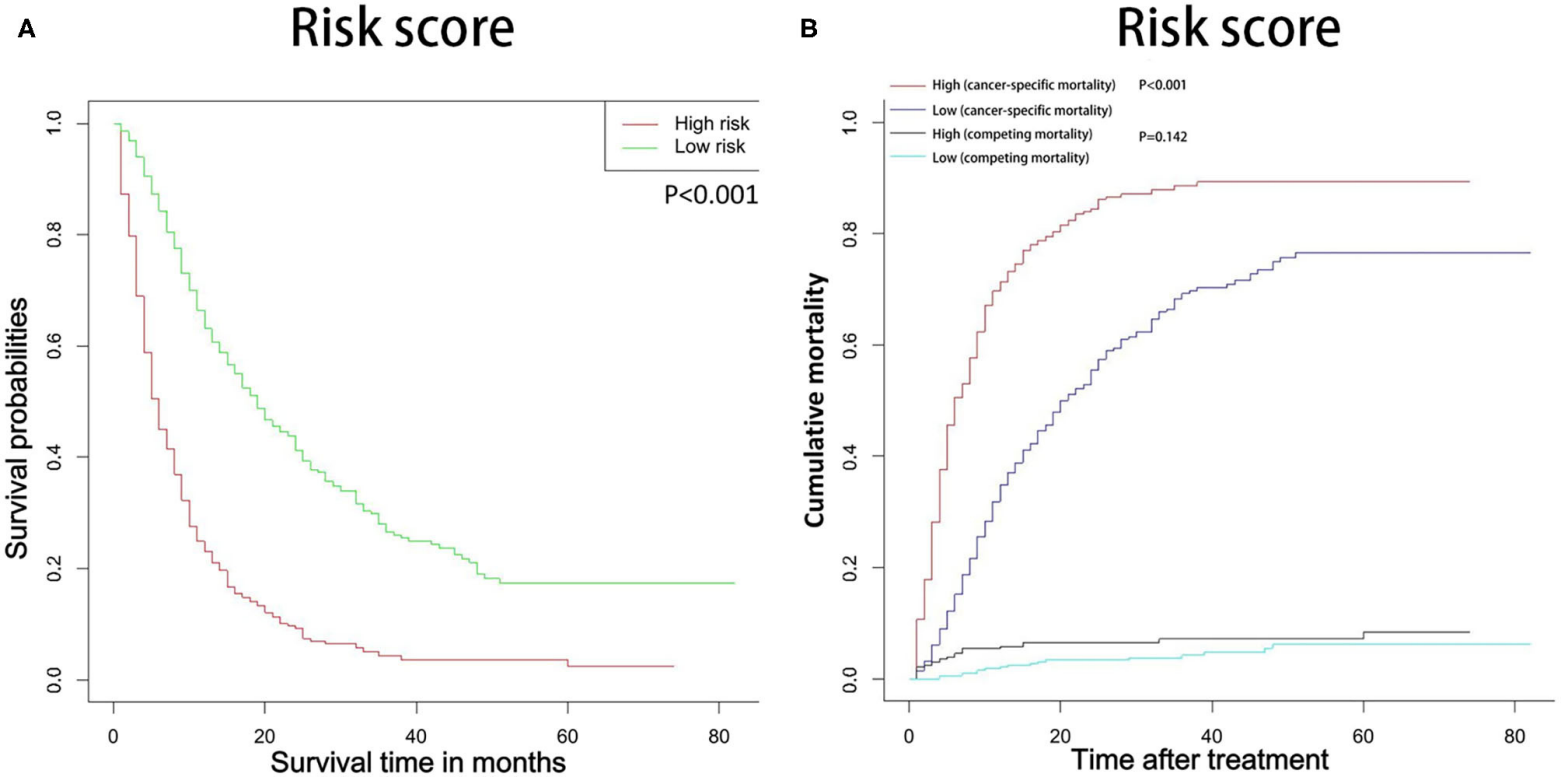
OS **(A)** and CSS **(B)** stratified by the risk levels of the nomogram-predicted survival probabilities. OS, overall survival; CSS, cancer-specific survival.

In this study, all patients with probability scores below or above the average score were divided into low-risk and high-risk groups. As shown in the Figure 8, compared with patients in the low-risk group, patients in the high-risk group had significantly lower survival rates and higher cancer-specific death rates.

## Discussion

In this study, we calculated the OS rate and competitive risk mortality of patients with advanced GBC patients who underwent radical resection. In this case, we established an effective prognostic model, which contains clinical features that the AJCC 8th TNM staging does not have.

Most studies have shown that the increase in age may have an impact on survival (23–25). In our study, most non-cancer-specific deaths occurred in elderly patients. It may be caused by the high comorbidity rate in elderly patients, which indicates that non-cancer-specific death is an extremely important competitive risk event for elderly patients (26). Therefore, it is very necessary to evaluate the surgical tolerance of elderly patients before surgery to reduce unnecessary non-cancer-specific deaths, and to pay attention to age when considering prognosis.

In our study of OS and CSS, the tumor differentiation, distant metastasis, radiotherapy and chemotherapy, and LODDS levels had a significant impact on survival. Similar to most studies, once a tumor had metastasized, it usually indicated a poor prognosis (14, 27). Even if the distant metastasis of the tumor is surgically removed, it is easy to recur, which will greatly affect the survival of the patient. The degree of tumor differentiation is an inherent characteristic of the tumor, and it is necessary to take it into consideration when considering the prognosis of the patient. In this study, we can saw that as the tumor grade changed from well to poor, the prognosis becomes worse. Like most studies (28, 29), the degree of tumor differentiation was also an independent risk factor affecting the prognosis of GBC patients. The advantage of the nomogram and the 8th AJCC TNM staging is that it can already add variables that are not included in the 8th AJCC TNM staging, and these variables are available in the clinic. Adjuvant therapy is becoming more and more important in the comprehensive treatment of tumors. In our study, both radiotherapy and chemotherapy had a significant impact on the prognosis of patients with advanced gallbladder cancer. Previous study (12) have shown that adjuvant therapy has benefited only in patients with stage II and higher grades, which is consistent with our results.

Lymph nodes are the most important metastasis route for GBC and an important factor affecting the prognosis after radical resection (30, 31), It may have a greater impact than T staging in patients with advanced focal lesions (32). In addition, the LODDS level also has a significant impact on the prognosis of GBC patients through univariate and multivariate analysis. This is different from the traditional N staging that only focuses on the number of lymph node metastases. LODDS also considers the number of negative lymph nodes to be removed. Previous study (13) have proved that LOODS has a significant impact on the prognosis of GBC patients, but it has a significant effect on the overall GBC patients, and what we have observed is more significant in advanced patients. Although LODDS is gradually recognized by clinicians and pathologists, there is still no uniform standard for the cut-off value of LODDS stratification.

We should consider non-cancer-specific death when evaluating the prognosis of decision-making and patient consultation, because this is the most important competitive risk in survival analysis. In this study, we included the factors of TNM staging and other tumor-related factors, which enhanced the ability to predict OS and CSS in advanced GBC patients. In addition, survival curve or competitive risk analysis demonstrates the use of nomograms to predict probabilistic survival rates or clear risk stratification for cancer-specific survival rates. As far as we know, this is the first study of OS and CSS nomograms for patients with advanced gallbladder cancer after surgery. At the same time, the nomogram also showed relatively high accuracy, with the C-index exceeding 0.7 in both the training cohort and the verification cohort. However, high accuracy does not necessarily indicate whether this prediction model has high clinical applicability (20, 33). The decision curve analysis can evaluate the clinical practical value of the prediction model by quantifying the net income of the prediction model according to the threshold probability (34). Therefore, we introduced DCA to evaluate the clinical utility of our nomogram, and the decision curve analysis confirmed the clinical validity of our nomogram in OS and CSS in patients with advanced gallbladder cancer after surgery.

Of course, our research also has some limitations. First of all, there are no relevant serological examinations in the SEER database and there are some surgical-related indicators, such as vascular invasion. These variables will be in our future research. Second, similar to other retrospective studies, both the development cohort and the validation cohort are affected by selection bias. Finally, our research only selected external verification from one hospital, and multi-center verification is needed to verify the accuracy of the nomogram. And our nomogram does not include all prognostic factors, and in clinical practice cannot always provide an accurate prognosis.

## Conclusion

In summary, based on the competitive risk analysis model and survival model established on the SEER database, this study established the estimated values of 1-, 2-, and 3-year OS and CSS for patients with advanced GBC after surgical resection. In addition, our nomograms showed relatively good performance, which may contribute to highly individualized patient management in clinical practice.

## Data Availability Statement

The raw data supporting the conclusions of this article will be made available by the authors, without undue reservation.

## Ethics Statement

Ethical review and approval was not required for the study on human participants in accordance with the local legislation and institutional requirements. Written informed consent for participation was not required for this study in accordance with the national legislation and the institutional requirements.

## Author Contributions

SZ was responsible for conception, design, quality control of this study, reviewed, and edited the manuscript. CY, ZH, QT, and KW performed the study selection, data extraction, statistical analyses, and were major contributors in writing the manuscript, and contributed to classification criteria discussion. CY, JW, and ZH participated in study selection and statistical analyses. All authors have read and approved the final version of the manuscript.

## Funding

This study was supported by the Research Project of Science and Technology from Healthy Committee of Jiangxi Province (No. 20204247) and Research Project of Science and Technology from Education Bureau of Jiangxi Province (No. 2020).

## Conflict of Interest

The authors declare that the research was conducted in the absence of any commercial or financial relationships that could be construed as a potential conflict of interest.

## Publisher's Note

All claims expressed in this article are solely those of the authors and do not necessarily represent those of their affiliated organizations, or those of the publisher, the editors and the reviewers. Any product that may be evaluated in this article, or claim that may be made by its manufacturer, is not guaranteed or endorsed by the publisher.
